# Impact of Recurrent Mitral Regurgitation on Left Ventricular Mass Regression and Cardiac Events following Mitral Valve Repair

**DOI:** 10.3390/jcm13010235

**Published:** 2023-12-30

**Authors:** Chih-Yao Chiang, Jih-Hsin Huang, Kuan-Ming Chiu, Jer-Shen Chen

**Affiliations:** 1 Department of Cardiovascular Surgery, Cardiovascular Center, Far Eastern Memorial Hospital, New Taipei City 220216, Taiwan; femh97737@femh.org.tw (C.-Y.C.); femh87919@femh.org.tw (J.-H.H.); 2 Division of Cardiovascular Surgery, Department of Surgery, School of Medicine, National Defense Medical Center, Taipei 114201, Taiwan

**Keywords:** mitral valve repair, recurrent mitral regurgitation, mass regression, reverse remodel

## Abstract

Background: Mitral valve regurgitation results in volume overload, followed by left ventricular remodeling. Variation of reverse remodeling following mitral repair influences the clinical outcomes. We aimed to evaluate the association between recurrent mitral regurgitation and mass regression following mitral valve repair and the impact on major adverse cardiovascular events. Methods: A retrospective cohort study was conducted on 164 consecutive patients with severe mitral regurgitation who underwent elective mitral valve repair. Subgroups were classified based on the presence of recurrent mitral regurgitation exceeding moderate severity. The hemodynamic parameters were evaluated according to geometry, mass, and function with Doppler echocardiography before and after surgery. Cox regression analysis was performed to evaluate the association between hemodynamics and mass regression and clinical outcomes. Results: The results for MR indicated 110 cases with non-recurrent MR and 54 with recurrent MR, along with 31 major adverse cardiovascular events. The tracked echocardiographic results revealed less reduction in dimension and volume, along with less mass regression in the recurrent MR subgroup. Significant differences were revealed in the relative change of the LV end-diastolic volume index and relative mass regression between subgroups. The relative change in the LVEDVI was proportionally correlated with relative mass regression. Cox regression analysis identified correlations with major adverse cardiovascular events, including suture annuloplasty, recurrent mitral regurgitation, tracked LV mass, relative LV mass regression, and systolic dysfunction. Conclusion: LV mass regression and relative change of the LV end-diastolic volume could be risk predictors of recurrent mitral regurgitation. The extent of LV mass regression is correlated with adverse cardiac events.

## 1. Introduction

Mitral regurgitation leads to left ventricular (LV) volume overload, causing atrial and ventricular dilatation and eccentric hypertrophy [1]. The time-dependent remodeling process involves adaptive hypertrophy and post-apoptotic fibrosis. Persistent left ventricular dilatation and dysfunction are associated with poorer outcomes [2]. LV hypertrophy is an independent risk predictor and associated with adverse cardiovascular events [3].

Mitral valve repair appears to be a promising option for mitral valve regurgitation. Its advantages encompass avoiding the need for anticoagulation, preserving the native mitral valve structure, maintaining left ventricular function, and promoting superior reverse remodeling. A broad spectrum of reconstructive techniques has been developed to treat complex MV pathology. The goals of mitral valve repair include recovery of an adequate coaptation area and normalizing LV volume and function. Recurrent mitral regurgitation and persistent hypertrophy are correlated with ventricular dysfunction [4]. The extent of LV mass regression serves as an indicator of reverse remodeling, and persistent hypertrophy impacts clinical outcomes [5].

Mitral valve repair corrects the regional structure and diminishes wall stress caused by volume overload, leading to a reduction in end-diastolic volume and pressure. Surgical correction initiates a process of reverse remodeling, characterized by a significant reduction in ventricular dilatation and enhanced diastolic function. Noticeable improvements in ventricular compliance and performance become apparent following mitral valve repair [6,7].

This study explores left ventricular reverse remodeling post-mitral valve repair. The hypothesis posits that reverse remodeling, manifested through mass regression and a relative alteration in end-diastolic volume, serves as a surrogate predictor for clinical outcomes. The objectives encompass three primary aims: firstly, to assess and compare the extent of reverse remodeling and functional outcomes among subgroups; secondly, to investigate the relationship between mass regression and recurrent mitral regurgitation; and finally, to investigate the relationship between hemodynamics and mass regression in relation to the incidence of major adverse cardiovascular events.

## 2. Materials and Methods

### 2.1. Study Population

From January 2017 to December 2021, a total of 164 symptomatic patients with severe mitral regurgitation underwent elective mitral valve surgery. All patients received baseline and tracked Doppler echocardiography. The inclusion criteria encompass individuals with degenerative mitral valve disease, who were candidates for mitral valve repair, with or without tricuspid valve annuloplasty [8,9]. The exclusion criteria encompass individuals with aortic valve or aortic disease, those who have undergone coronary artery bypass grafting, individuals with ischemic mitral regurgitation, and cases for which tracked echocardiographic data are unavailable. Patients were categorized based on the presence of recurrent mitral regurgitation, defined as MR larger than moderate [8,9]. All patients were examined using Doppler echocardiography routinely before surgery and had a tracked follow-up exam at least 6 months after at the outpatient department. These evaluations were performed without inotropic support status. The assessment time was tracked, and the median interval was 13.6 months (95% CI 11.7 to 15.0 months). Ultrasound examinations were conducted using the Philips iE33 ultrasound system from Philips Medical Systems. All data were retrieved from electronic medical records and from clinic visits. The study was approved by the Institutional Review Board of the Far Eastern Memorial Hospital (approval number: 111256-E).

### 2.2. Severity of Mitral Valve Regurgitation

Severity of MR was graded based on the current American Society of Echocardiography recommendations of effective regurgitant orifice area (EROA): mild (<0.2 cm^2^), moderate (0.2–0.29 cm^2^), moderate to severe (0.3–0.39 cm^2^), and severe (>0.40 cm^2^); regurgitant volume: mild (<30 mL), moderate (30–44 mL), moderate to severe (45–59 mL), and severe (>60 mL); and regurgitant fraction: mild (<30%), moderate (30–39%), moderate (40–49%), and severe (>50%). Integrative assessment included a final determination of MR according to the following grades: 1 = mild; 2 = moderate; 3 = moderate to severe; and 4 = severe. We characterize recurrent mitral regurgitation as the presence of moderate to severe regurgitation with a grading of MR ≥ 3 [8,9].

### 2.3. Repair Technique

The surgical procedure utilized cardiopulmonary bypass with aortic and bicaval cannulation, maintaining moderate hypothermia through either a mini thoracotomy approach or sternotomy. Mitral valve repair was executed using a combination of techniques, including ring annuloplasty [10], modified Kay–Wooler annuloplasty [11], artificial chordal implantation [12], segmental wedge resection [13], commissuroplasty [11], and the edge-to-edge technique [14].

### 2.4. Surgical Outcomes

#### 2.4.1. Hospital Results

Systolic anterior movement (SAM) is indicative of obstruction of the left ventricular outflow tract [15]. Exploratory thoracotomy is necessitated by massive mediastinal bleeding. Transient pacemaker support is provided during the postoperative period in the intensive care unit (ICU). Low cardiac output is defined as the maximal need for inotropic agent support, specifically a Vasoactive-Inotropic Score (VIS) exceeding 10 after surgery [16].

$$\mathrm{VIS}\;\mathrm{score}=(\mathrm{Dopamine}+\;\mathrm{Dobutamine}+\;100\cdot \mathrm{Epinephrine}+\;100\cdot \mathrm{Norepinephrine}\;\;\;\;\;\;\;\;\;\;\;\;\;\;\;\;\;\;\;\;\;\;\;\;\;\;\;\;\;\;\;\;\;\;\;+\;50\cdot \mathrm{Levosi}\;\mathrm{mendan}+\;10\cdot \mathrm{milrinone}+\;10000\cdot \mathrm{Vasopressin})\;\mu g\cdot {kg}^{-1}\cdot {min}^{-1}$$

We define the immediate residual MR after repair according to the immediate TEE report after cardiopulmonary bypass [8,9]. In-hospital mortality was defined as all-cause death before discharge in the same hospitalization.

#### 2.4.2. Follow-Up Results

Composite cardiac adverse events encompass all-cause death, myocardial infarct, stroke, hospitalization for heart failure, and reoperation for mitral valve surgery that occurred during the follow-up period. Survivors were monitored at the outpatient department and underwent echocardiographic assessments at our hospital.

### 2.5. Echocardiographic Parameters

#### 2.5.1. LV Geometry Parameters

Geometric measurements, including the LV end-diastolic diameter (LVEDD), LV end-systolic diameter (LVESD), end-diastolic septal thickness (IVSd), and end-diastolic inferolateral wall thickness (ILWd), were determined using M-mode echocardiography. The left ventricular end-diastolic volume (LVEDV) and end-systolic volume (LVESV) were derived from the apical two-chamber and four-chamber views utilizing the Teichholz formula. The biplane Simpson formula was not employed due to constraints within the echocardiography room. LV mass (LVM) was calculated using the corrected American Society of Echocardiography formula as follows:

$$\mathrm{LV}\;\mathrm{Mass}=0.8\;\times \;[1.04\;\times \;[(\mathrm{IVSd}\;+\;\mathrm{LVEDD}\;+\;\mathrm{ILWd}{)}^{3}\;-\;{\mathrm{LVEDD}}^{3}]+0.6$$

The indexed LV mass was indexed by body surface area.

The relative wall thickness (RWT) ratio was calculated as:

$$\mathrm{Relative}\;\mathrm{wall}\;\mathrm{thickness}\;\mathrm{ratio}=\frac{2\times end-diastolic\;inferolateral\;wall\;thickness}{LV\;end\;diastolic\;dimension}$$

Definition of remodel mode: Hypertrophy means LV mass exceeds cutoff value:

$$\mathrm{Female}\;>\;95\;g\cdot m^{-2},\;\mathrm{Male}\;>\;115\;g\cdot m^{-2}.$$

Remodel Mode: Concentric means RWT > 0.42, Eccentric RWT < 0.42 [17].

$$\mathrm{Relative}\;\mathrm{change}\;(\%)=\frac{postoperative\;parameter-preoperative\;parameter\;}{preoperative\;parameter}\times 100$$

#### 2.5.2. LV Systolic Function

$$\mathrm{LV}\;\mathrm{ejection}\;\mathrm{fraction}=\frac{LV\;end\;diastolic\;volume-LV\;end\;systolic\;volume}{LV\;end\;diastolic\;volume}\times 100$$

$$Strok\;evolume=\left[\pi \times {\left(\frac{LVOT}{2}\right)}^{2}\right]\times {VTI}_{LVOT}$$

Stroke volume index was stroke volume indexed by body surface area.

#### 2.5.3. LV Diastolic Function

The assessment of diastolic function involved parameters of trans-mitral inflow, including the peak velocities of the E wave (representing early diastole), the A wave (representing late diastole), the ratio of E/A, the deceleration time of the E wave, and the e′ wave (early diastolic lateral mitral annular velocities). The E/e′ ratio was calculated as the ratio between the E velocity and e′ velocity. The grading of diastolic dysfunction was determined based on guidelines [18]. The LV end-diastolic pressure was estimated using LVEDP≅ 1.24 $\times \frac{E}{e\prime }$ +1.9 [19].

### 2.6. Statistical Analysis

Continuous variables are presented as the median and at the 95% confidence interval in the Mann–Whitney test for independent samples. Paired samples are used in the Wilcoxon test to compare hemodynamic parameters. Categorical variables are presented as frequencies, and compared using the Fisher exact test. ROC curve analysis is employed to identify the optimal cutoff value for predictors in MACEs. Multivariate linear regression analysis is used to identify the independent hemodynamics with LV mass regression. The multivariate Cox proportional hazards model is used to assess the correlation between hemodynamics and MACEs of interest, with the results presented as a hazard ratio (HR) and at the 95% confidence interval (CI). The regression models are adjusted for age, hypertension, and systolic dysfunction (preoperative LVEF < 55%). The cumulative probability of death is estimated using the Kaplan–Meier method, and survival is compared between two subgroups using the log rank test. All tests are two-tailed, and the level of statistical significance is set at *p* < 0.05. Statistical analyses were conducted using the MedCalc. software v22.016.

## 3. Results

In this comprehensive analysis, we included 164 patients who underwent mitral valve repair, with a median follow-up time of 37 months (95% CI 32 to 40 months). Notably, there were no perioperative deaths among the surgical results. Tracked mitral regurgitation revealed 110 cases with MR < 3 and 54 with MR ≥ 3. Over the follow-up period, 41 major adverse cardiovascular events (MACEs) were recorded, including 1 myocardial infarct due to infective endocarditis, 7 cases of cerebrovascular accidents, 30 instances of heart failure necessitating rehospitalization, and 15 reoperations for mitral valve surgery.

### 3.1. Patient Characteristics

The mean age of the total cohort was 58.3 ± 11.9 years old, with 67% being male. No significant differences were observed among subgroups. Common comorbidities included hypertension (44%), dyslipidemia (26%), and coronary artery disease (26%). There were no discernible differences among subgroups, except for the presence of atrial fibrillation. Ventricular dilatation, as indicated by the left ventricular end-diastolic diameter (LVEDD) exceeding 55 mm, was noted in 52% of cases. Ventricular function was classified as systolic dysfunction in 5% and diastolic dysfunction in 79%, with Grade 1 dysfunction in 16%, Grade 2 in 37%, and Grade 3 in 27% of cases. Remodeling patterns included eccentric remodeling in 55% of cases and concentric hypertrophy in 26%. Pulmonary hypertension greater than moderate was observed in 29% of the cohort (Table 1).

### 3.2. Operative Characteristics

The analysis of valve pathology revealed annular dilatation in 79% of cases, involvement of a cleft segment in 20% of cases, and chordae prolapse/rupture, with the anterior mitral leaflet (AML) accounting for 15% and the posterior mitral leaflet (PML) for 74%. The repair techniques were generally similar, differing only in ring annuloplasty and atrial ablation. Following repair, the distribution of residual MR was as follows: trivial in 32 (20%) cases, mild in 28 (17%) cases, and moderate in 1 (1%) case. Postoperative morbidity included systolic anterior motion in 2 (2%) cases, temporary pacing in 25 (15%) cases, low cardiac output in 40 (24%) cases, and exploratory thoracotomy for mediastinal bleeding in 7 (4%) cases. Ring annuloplasty was used in 110 cases (67%), with various types such as Physio II (73%), Cosgrave (19%), and Memo3D (8%). Suture annuloplasty was employed in 54 cases (33%) (Table 2).

### 3.3. Varying Reverse Remodeling following Mitral Valve Repair

At baseline, there were no substantial differences in dimension, volume, LV mass, and function among subgroups. Through paired comparisons across the entire cohort following repair, we observed a reduction in LV dimension, volume, and mass, accompanied by enhanced diastolic function, with similar outcomes reflected in subgroups. The improvements in diastolic function and end-diastolic pressure were evident in both subgroups. However, the tracked results revealed less reduction in dimension and volume, along with less mass regression, particularly in the recurrent MR subgroup. This same phenomenon was observed in diastolic function in the tracked echo data, as detailed in Table 3.

### 3.4. Correlation of Mass Regression with Clinical Outcomes

**Figure 1:**
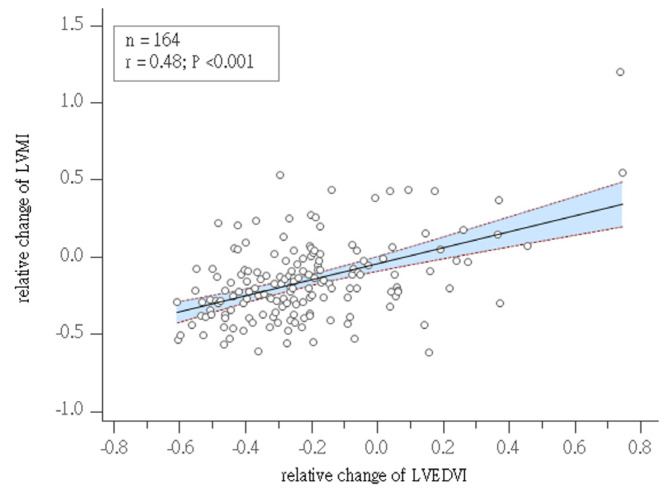
The relative change of LVMI proportionally correlated with relative LV mass regression.

**Figure 2:**
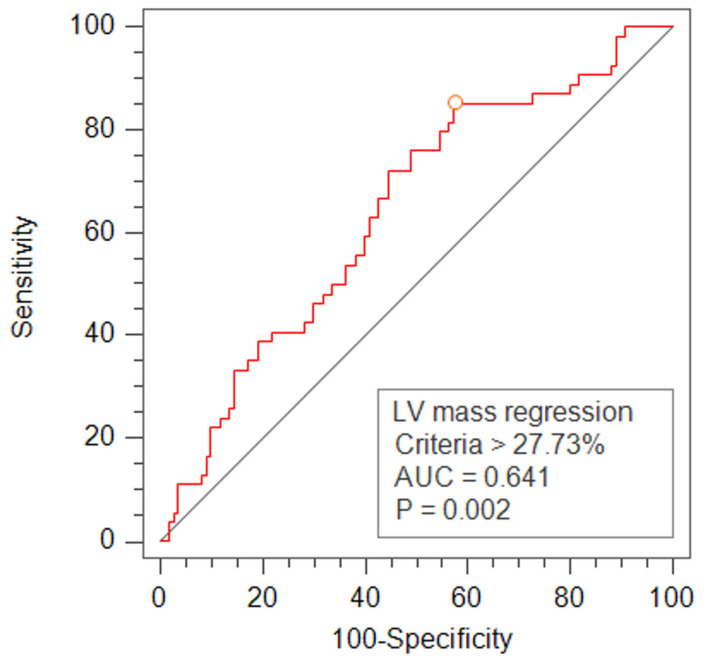
The receiver operating characteristic (ROC) curve for LV mass regression. The red line means ROC curve, The AUC means area under the ROC curve.

**Figure 3:**
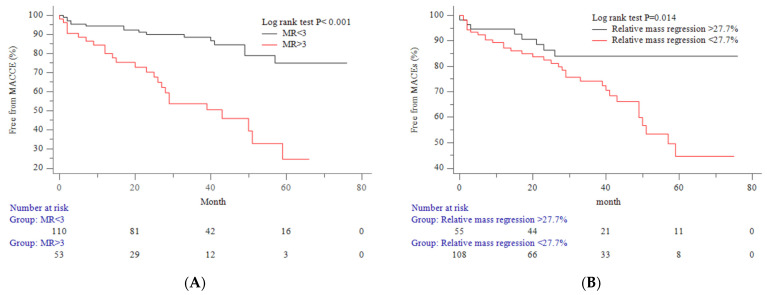
The freedom from events rate of MACEs for patients with MR < 3 and MR ≥ 3 is shown in (**A**). The corresponding optimal cutoff of relative LVM regression was −27.7%, and the freedom from events rate against the cutoff is shown in (**B**).

As illustrated in Table 4, significant differences emerged in the relative change of the left ventricular end-diastolic volume index (LVEDVI) and relative mass regression between subgroups. Notably, multivariate regression analysis, detailed in Table 5, indicated that only the relative change in the LVEDVI was associated with relative mass regression. Figure 1 visually represents this association between the relative change in the LVEDVI and relative mass regression. Employing receiver operating characteristic (ROC) analysis, a cutoff value of −27.7% for relative mass regression was determined (Figure 2). The cumulative events rate in subgroups, categorized by recurrent mitral regurgitation and relative change of mass regression, was visualized using Kaplan–Meier curves (Figure 3A,B). Risk predictors for recurrent MR included suture annuloplasty. Risk predictors for major adverse cardiovascular events included recurrent mitral regurgitation, follow-up left ventricular (LV) mass index, follow-up left ventricular diastolic volume index, relative LV mass regression, and systolic dysfunction, as illustrated in Table 6.

## 4. Discussion

The spectrum of post-repair recurrent mitral regurgitation, coupled with variations in reverse remodeling, significantly impacts ventricular function and clinical outcomes. Substantial differences were observed in both the relative changes of end-diastolic volume and LV mass regression between subgroups. The reduction in end-diastolic volume demonstrated a proportional correlation with mass regression. The risk predictors in MACEs encompass recurrent MR, tracked LV mass index, tracked LVEDVI, relative mass regression, and systolic dysfunction.

### 4.1. Valve Pathology and Adaptive Hypertrophy to Volume Overload

The valve pathology leading to inadequate coaptation area includes mitral annular disjunction, chordae prolapse or rupture, and leaflet redundancy or disruption [20]. The transformed mitral structures initiate a time-dependent global transitional remodeling. Volume overload increases wall stress and influences the NO-cGMP-PKG-titin signaling pathway [6]. This modulation serves as an insufficient brake on the prohypertrophic stimulus of HFpEF-related LV hypertrophy. Additionally, the mismatch between wall stress and coronary flow reserve contributes to myocardial ischemia and adaptive hypertrophy [6]. The myocardial remodeling in response to volume overload is characterized by eccentric hypertrophy, signifying impaired compliance and diastolic dysfunction. This process involves adaptive sarcomere hypertrophy and irreversible interstitial fibrosis, albeit at the expense of mechanical efficiency [7,21]. Resultant myocardial fibrosis leads to maladaptive decompensation and implications for cardiac events and mortality [2].

### 4.2. Facilitating Reverse Remodeling through Mitral Valve Repair

Mitral valve repair is prioritized over replacement, given its superior ability to restore ventricular function while preserving the integrity of the mitral valve complex [22]. Repair enhances the achievement of a larger effective orifice area, lowers the trans-mitral pressure gradient, and minimizes the risks linked to prosthetic structural valvular deterioration. The primary goal of repair is to reconstruct an adequate coaptation area and ensure durability. Utilizing evidence-based techniques such as neo-chordae implantation, saddle-ring annuloplasty, and edge-to-edge approximation contributes to reducing stress on leaflets and chordae tendineae, thereby enhancing the repair durability [23,24]. Mitral valve repair modifies the deformed mitral structure and alters force vectors within the LV chamber. Following mitral repair, the chamber adapts to the reduced preload and afterload, the acquired larger effective orifice area, and reduced preload, along with diminishing ineffective work due to volume overload. These alterations influence wall stress in the left atrium and ventricle, as well as the NO-cGMP-PKG-Titin signal pathway associated with adaptive hypertrophy. Reverse remodeling, marked by mass regression and a reduction in end-diastolic volume, is observed. The improved ventricular performance, achieved through enhanced compliance, reduced energy consumption, and optimized coupling between the mitral and ventricular functions, leads to enhanced mechanical efficiency [4].

### 4.3. Relation between Relative Change of LVEDVI and LVMI Regression

Variations in reverse remodeling are evident after mitral valve repair. Yokoyama et al. [25] achieved reverse remodeling with significant volume reduction of the LV chamber in transcatheter mitral valve repair. Shyu et al. [26] disclosed that MV repair led to reduction of chamber size and LV mass regression. Seldrum et al. [27] revealed that significant LV reverse remodeling after MV repair and LV volumes were the most powerful predictors for adverse remodeling. In this study, we observed a substantial reduction in end-systolic volume, end-diastolic volume, stroke volume, and end-diastolic pressure based on the chamber volume change. In the end-diastolic pressure–volume relationship, the curve shifts leftward, signifying a decrease in both end-diastolic pressure and end-diastolic volume. This adaptive alteration improves compliance and reduces workload. However, in the recurrent MR subgroup, it indicates a smaller reduction in ESVI, EDVI, SVI, and EDP. That means larger volume overload and filling pressure, resulting in a less pronounced leftward shift in the EDPVR and increased ineffective energy consumption. Additionally, the recurrent MR subgroup exhibited less mass regression.

### 4.4. Repair Technique and Variability of Reverse Remodeling Impact on Clinical Outcomes

Suture annuloplasty poses a greater risk of recurrent mitral regurgitation compared to saddle-ring annuloplasty. In the early stages, surgeons opted for this method if there was no apparent annular dilatation observed directly or by transesophageal echocardiography evaluation. While suture annuloplasty preserves annular flexibility and folding function, it fails to restore the natural saddle conformation. The persistent higher stress leads to the rupture of the suture site at the free edge of the leaflet, contributing to relatively early recurrence. In contrast, the saddle ring offers distinct advantages by securely anchoring the annulus to the saddle-like frame, maintaining a systolic saddle conformation, and reducing stress exponentially on the leaflet and chordae tendineae. Tracked echocardiographic findings and observations during redo operations reveal that chordae elongation or rupture predominantly occurs in the posterior leaflet, specifically at the P2 segment. The saddle-like ring proves superior to suture annuloplasty [23].

Eccentric hypertrophied myocardium is characterized by reduced contractility and increased stiffness, along with increased energy consumption and diastolic dysfunction [3]. Post-apoptotic fibrosis resulting from chronic volume overload is characterized by progressive and irreversible decompensated structural and functional alterations [2]. In this study, the relative change of end-diastolic volume can serve as a marker for reverse remodeling, and the extent of mass regression is correlated with adverse events. Reductions including in the LVEDV and the extent of volume overload promote mass regression and the EDPVR shift to the left. Improvements in diastolic dysfunction and LV end-diastolic pressure were verified in this study. Imasaka et al. [4] revealed early MV repair for severe mitral regurgitation improves LV mass regression and ventricular performance. Stulak et al. [28] disclosed early mitral valve repair for leaflet prolapse optimizes LV reverse remodeling and recovery to normal LV function. In our study, it was evident that the tracked left ventricular end-diastolic volume index (LVEDVI) and left ventricular mass index (LVMI) serve as potential risk factors for recurrent mitral regurgitation. In the subgroup without recurrent MR, there was a noticeable improvement in compliance and a reduction in end-diastolic pressure. Conversely, in the subgroup with recurrent MR, there was less mass regression and persistent hypertrophy. The relative change in left ventricular end-diastolic volume is directly proportional to relative left ventricular mass regression. This implies that surgical modifications to the mitral valve complex, aimed at improving coaptation and reducing regurgitation, play a significant role in achieving more substantial reverse remodeling and enhancing clinical outcomes.

#### 4.4.1. Clinical Implication

The study elucidates the relationship between the volume and mass of the LV chamber and recurrent mitral regurgitation. The relative change of left ventricular end-diastolic volume index was significantly proportional to relative mass regression. The presence of recurrent MR, along with persistent volume overload and inadequate reverse remodeling, is associated with adverse cardiac events.

#### 4.4.2. Study Limitation

Firstly, this study was a nonrandomized retrospective investigation with a small number of patients. Secondly, the calculation of ventricular volume using the Teichholz formula, and the quality of the echocardiographic assessment is observer dependent. Finally, the surveillance interval of the echocardiographic data of the 164 patients varied. LV mass regression is a complex phenomenon that is influenced by patient characteristics and duration of volume overload. Residual hypertrophy after a reduction of LV pressure overload may be due to irreversible changes in interstitial fibrosis owing to long-term disease.

## 5. Conclusions

Left ventricular mass regression and the relative change of left ventricular end-diastolic volume could be risk predictors of recurrent mitral regurgitation. The extent of LV mass regression is correlated with adverse cardiac events. These data reinforce the notion that improved mass regression and a reduction in left ventricular end-diastolic volume contribute to enhanced cardiac performance and better clinical outcomes. However, long-term assessments of left ventricular performance and left ventricular mass regression are warranted for a comprehensive understanding.

## Figures and Tables

**Table 1:** Baseline characteristics of demography, comorbidity, remodel mode, and dysfunction according to recurrent MR.

	Variable	Total (N = 164)	MR < 3 (N = 110)	MR≥ 3 (N = 54)	*p*
	Age	58.3 ± 11.9	58.6 ± 12.7	57.6 ± 10.1	0.596
	Male	109 (67)	77 (70)	32 (59)	0.172
Comorbidity	Hypertension	72 (44)	50 (45)	22 (41)	0.569
	Dyslipidemia	43 (26)	31 (28)	12 (22)	0.416
	CAD	42 (26)	29 (26)	13 (24)	0.753
	DM	31 (19)	20 (18)	11 (20)	0.737
	CKD	18 (11)	15 (14)	3 (6)	0.183
	COPD	9 (5)	4 (4)	5 (9)	0.139
	CVA	8 (5)	7 (6)	1 (2)	0.209
	AF	48 (29)	40 (36)	8 (15)	0.005
	LVEDD > 55 mm	86 (52)	55 (50)	31 (57)	0.408
Dysfunction	Systolic	8 (5)	4 (4)	4 (7)	0.441
	LVEF: 40–55%	4 (2)	2 (2)	2 (4)	0.599
	LVEF < 40%	4 (2)	2 (2)	2 (4)	0.599
	Diastolic	130 (79)	85 (77)	45 (83)	0.418
	Gr1	26 (16)	18 (16)	8 (15)	1.000
	Gr2	60 (37)	41 (37)	19 (35)	0.864
	Gr3	44 (27)	26 (24)	18 (33)	0.195
Remodel Mode	Normal	24 (15)	17 (15)	7 (13)	0.815
	Concentric remodel	7 (4)	5 (5)	2 (4)	1.000
	Concentric hypertrophy	43 (26)	33 (30)	10 (19)	0.134
	Eccentric hypertrophy	90 (55)	55 (50)	35 (65)	0.095
Pulmonary Hypertension	No	57 (35)	43 (39)	14 (26)	0.117
	Mild	60 (37)	40 (36)	20 (37)	1.000
	Moderate	28 (17)	18 (16)	10 (19)	0.826
	Severe	19 (12)	9 (8)	10 (19)	0.069

Abbreviations: CAD, coronary artery disease; DM, diabetes mellitus; CKD, chronic kidney disease; COPD, chronic obstructive pulmonary disease; CVA, cerebrovascular accident; AF, atrial fibrillation; LVEDD, left ventricular end-diastolic dimension; LVEF, left ventricular ejection fraction. Data are expressed as frequency (percentage) or mean ± standard deviation.

**Table 2:** Operative characteristics: mitral valve pathology, repair technique, and outcomes according to recurrent MR.

		Total (N = 164)	MR < 3 (N = 110)	MR≥ 3 (N = 54)	*p*
Access	Mini thoracotomy	155 (95)	106 (96)	49 (91)	0.157
Valve pathology	Annular dilatation	130 (79)	90 (82)	40 (74)	0.252
	Cleft segment	33 (20)	25 (23)	8 (15)	0.236
	Chordae prolapse/rupture				
	AML	30 (18)	20 (18)	10 (19)	1.000
	PML	121 (74)	72 (65)	39 (72)	0.478
Repair technique	Annuloplasty ring	110 (67)	81 (74)	29 (54)	0.011
	Neo-chordae implantation	128 (78)	84 (76)	44 (81)	0.485
	Segment approximate	35 (21)	25 (23)	10 (19)	0.538
	Commissure approximate	27 (16)	17 (15)	10 (19)	0.620
	Wedge resection	4 (2)	3 (3)	1 (2)	0.734
Combined procedure	Atrial ablation	46 (28)	37 (34)	9 (17)	0.023
	Tricuspid valve annuloplasty	43 (26)	25 (23)	18 (33)	0.117
Residual MR	No	103 (63)	73 (66)	30 (56)	0.229
	Trivial	32 (20)	20 (18)	12 (22)	0.537
	Mild	28 (17)	16 (15)	12 (22)	0.270
	Moderate	1 (1)	1 (1)	0 (0)	1.000
Morbidity	SAM	3 (2)	2 (2)	1 (2)	1.000
	Temporary pacing	24 (15)	19 (17)	5 (9)	0.240
	Low cardiac output	40 (24)	29 (26)	11 (20)	0.445
	Exploratory thoracotomy	7 (4)	5 (5)	2 (4)	1.000

Abbreviations: AML, anterior mitral leaflet; PML, posterior mitral leaflet; SAM, systolic anterior movement. Data are expressed as frequency (percentage); *p* indicates the comparison between subgroups according to recurrent MR.

**Table 3:** Comparisons of geometry and hemodynamics before and after MV repair according to recurrent MR.

		Baseline	Follow-up		Baseline			Follow-up				
	Variable	Total, N = 164	Total, N = 164	*p*	MR < 3, N = 110	MR≥ 3, N = 54	*p*	MR < 3, N = 110	MR≥ 3, N = 54	*p*	*p* *	*p* †
Geometry	LVESD, cm	3.3 (3.2, 3.4)	3.0 (2.9, 3.1)	<0.001	3.3 (3.2, 3.5)	3.3 (3.2, 3.5)	0.460	2.9 (2.8, 3.0)	3.1 (2.9, 3.3)	0.007	<0.001	0.003
	LVEDD, cm	5.5 (5.4, 5.6)	4.8 (4.7, 4.9)	<0.001	5.5 (5.3, 5.6)	5.6 (5.4, 5.7)	0.247	4.76 (4.65, 4.90)	5.11 (4.84, 5.27)	<0.001	<0.001	<0.001
	LVESVI, mL·m^−2^	25.5 (24.2, 27.7)	20.7 (18.3, 21.8)	<0.001	24.9 (23.5, 28.3)	26.4 (24.5, 30.3)	0.226	18.5 (17.0, 21.1)	22.7 (20.0, 25.9)	0.002	<0.001	0.003
	LVEDVI, mL·m^−2^	86.9 (80.7, 91.9)	62.3 (60.3, 65.7)	<0.001	82.7 (78.1, 91.9)	87.8 (81.6, 98.1)	0.083	60.2 (56.4, 61.9)	76.4 (65.1, 79.8)	<0.001	<0.001	<0.001
	LVMI, g·m^−2^	145 (137, 149)	114 (106, 124)	<0.001	142 (130, 148)	148 (138, 164)	0.080	105 (97, 113)	133 (128, 143)	<0.001	<0.001	0.003
	MVA, cm^2^	4.1 (4.0, 5.0)	2.6 (2.4, 2.9)	<0.001	4.0 (4.0, 5.0)	4.9 (4.0, 5.0)	0.408	2.6 (2.4, 2.9)	3.0 (2.6, 3.4)	0.078	<0.001	<0.001
Systolic	SVI, mL·m^−2^	58.2 (55.0, 61.9)	42.1 (40.6, 43.7)	<0.001	57.3 (53.9, 61.5)	59.8 (55.0, 65.6)	0.214	40.6 (38.6, 42.2)	48.5 (42.5, 52.4)	<0.001	<0.001	<0.001
	LVEF, %	68.8 (66.9, 70.2)	67.7 (66.5, 68.7)	0.334	68.9 (66.5, 71.1)	68.6 (66.44, 70.7)	0.782	67.8 (66.3, 68.8)	67.6 (65.6, 70.1)	0.704	0.617	0.312
Diastolic	DT, ms	190 (180, 197)	246 (229, 261)	<0.001	192 (176, 197)	190 (168, 204)	0.841	261 (233, 282)	227 (197, 247)	0.069	<0.001	0.001
	e′, cm·s^−1^	8.4 (7.6, 9.0)	10.0 (9.9, 10.0)	<0.001	8.6 (7.9, 10.0)	7.4 (6.7, 8.9)	0.061	10.0 (10.0, 10.2)	10.0 (9.0, 10.0)	0.240	<0.001	<0.001
	E/e′, ratio	14.6 (13.6, 15.9)	12.2 (11.1, 13.2)	<0.001	14.2 (12.6, 15.2)	14.9 (13.7, 19.3)	0.074	11.0 (10.3, 12.2)	13.8 (12.4, 15.6)	0.011	<0.001	0.047

Abbreviations: LVESD, left ventricular end-systolic dimension; LVEDD, left ventricular end-diastolic dimension; LVESVI, left ventricular end-systolic volume index; LVEDVI, left ventricular end-diastolic volume index; LVMI, left ventricular mass index; MVA, mitral valve area; SVI, stroke volume index; LVEF, left ventricular ejection fraction; E, early diastolic trans-mitral flow velocity; DT, trans-mitral deceleration time; e′, early lateral mitral annulus tissue doppler velocity. *p* * indicates comparison between MR < 3 before and after MV repair; *p* † indicates comparison between MR ≥ 3 before and after MV repair.

**Table 4:** Comparison of relative change in hemodynamics between subgroups according to recurrent MR.

Relative Change, %	MR< 3 (N = 110)		MR≥ 3 (N = 54)		*p*
∆ LVESVI	−22.8	−31.7, −16.0	−17.3	−24.5, −2.0	0.059
∆ LVEDVI	−27.2	−32.3, −24.4	−20.3	−26.5, −17.1	0.016
∆ LVMI	−22.7	−28.2, −17.1	−12.8	−18.8, −2.8	0.003
∆ MVA	−40.6	−45.9, 34.5	−40.2	−51.5, 26.6	0.735
∆ SVI	−26.9	−35.7, −20.9	−19.9	−29.9, −14.0	0.209
∆ LVEF	−0.8	−4.3, 2.7	−1.6	−4.7, 1.1	0.740
∆ DT	46.2	27.2, 58.8	23.4	10.0, 40.8	0.100
∆ e′	−16.0	−23.1, −2.6	−15.3	−29.9, −4.0	0.883
∆ E/e′	22.2	3.2, 37.3	17.5	−8.9, 35.2	0.713

Data are expressed using the median and 95% confidence interval. ∆, relative change.

**Table 5:** Correlation between relative change of hemodynamics and relative mass regression.

Relative Change, %	Univariate			Multivariate		
	β	r	*p*	β	r	*p*
∆ LVESVI	0.188	0.31	0.001			
∆ LVEDVI	0.520	0.48	<0.001	0.52	0.48	<0.001
∆ MVA	0.028	0.04	0.659			
∆ SVI	0.326	0.41	<0.001			
∆ LVEF	0.036	0.04	0.647			
∆ DT	−0.001	0.01	0.906			
∆ e′	−0.037	0.05	0.566			
∆ E/e′	0.040	0.12	0.127			

Abbreviations are the same as described in Table 3 and Table 4; ∆, relative change. β indicates regression coefficient; r indicates correlation coefficient.

**Table 6:** Association between operative variables and recurrent MR ≥ 3; correlation of follow-up variables and relative mass regression with the risk of MACEs.

	Univariate			Multivariate		
	HR	CI, 95%	*p*	HR	CI, 95%	*p*
Recurrent MR ≥ 3						
Suture annuloplasty	2.08	1.21, 3.57	0.008	2.88	1.59, 5.22	0.001
Ablation	0.44	0.21, 0.90	0.024			
Residual MR > 1	1.61	0.93, 2.76	0.088			
MACEs						
MR ≥ 3	4.40	2.34, 8.28	<0.001	5.04	2.65, 9.58	<0.001
LVMI, 10 g·m^−2^	1.14	1.07, 1.21	<0.001	1.09	1.01, 1.17	0.023
LVESVI, mL·m^−2^	1.02	1.01, 1.04	<0.001			
LVEDVI, mL·m^−2^	1.02	1.01, 1.03	0.010	1.01	1.00, 1.03	0.032
SVI, mL·m^−2^	1.00	0.97, 1.02	0.774			
LVEF, %	0.96	0.94, 0.98	<0.001	0.96	0.94, 0.98	<0.001
DT, ms	1.00	0.99, 1.00	0.396			
e′, cm·s^−1^	0.91	0.79, 1.04	0.153			
E/e′, ratio	1.03	0.99, 1.07	0.076			
Relative change LVMI, %	2.21	1.27, 5.69	0.039	2.69	1.06, 6.86	0.038

Abbreviations: MR ≥ 3 indicates recurrent MR; LVMI %, relative change of left ventricular mass; LVESVI, left ventricular end-systolic volume index; LVEDVI, left ventricular end-diastolic volume index; SVI, stroke volume index; DT, deceleration time of mitral inflow; E/e′, mitral inflow early velocity/tissue doppler lateral mitral annulus; MACEs, major adverse cardiovascular events; HR, hazard ratio; CI, confidence interval. Adjusted for age and preoperative LVEF, LVEDVI, pulmonary hypertension ≥ moderate.

## Data Availability

The data presented in this study are available on request from the corresponding author. The data are not publicly available due to privacy and ethical reasons.

## Abbreviations

EOA	effective orifice area
LVEDP	LV end-diastolic pressure
LVESVI	LV end-systolic volume index
LVEDVI	LV end-diastolic volume index
LVMI	LV mass index
MPG	Mean pressure gradient
MR	Mitral regurgitation
MVR	Mitral valve repair
MACCE	Major adverse cardiac and cerebral events
RWT	Relative wall thickness
